# Unique Thermal Properties of Clothing Materials

**DOI:** 10.1002/gch2.201800082

**Published:** 2019-02-27

**Authors:** Ning Pan

**Affiliations:** ^1^ Department of Biological and Agricultural Engineering University of California at Davis CA 95616 USA

**Keywords:** clothing, fabrics, heat transfer, thermal comfort

## Abstract

Cloth wearing seems so natural that everyone is self‐deemed knowledgeable and has some expert opinions about it. However, to clearly explain the physics involved, and hence to make predictions for clothing design or selection, it turns out to be quite challenging even for experts. Cloth is a multiphased, porous, and anisotropic material system and usually in multilayers. The human body acts as an internal heat source in a clothing situation, thus forming a temperature gradient between body and ambient. But unlike ordinary engineering heat transfer problems, the sign of this gradient often changes as the ambient temperature varies. The human body also perspires and the sweat evaporates, an effective body cooling process via phase change. To bring all the variables into analysis quickly escalates into a formidable task. This work attempts to unravel the problem from a physics perspective, focusing on a few rarely noticed yet critically important mechanisms involved so as to offer a clearer and more accurate depiction of the principles in clothing thermal comfort.

## 1 Introduction

Cloth can be defined as a designed 3D textile structure made of 2D fabrics that are networks of yarns formed in turn by spinning fibers. Cloth wearing is a behavior unique to human beings of virtually all societies, and it seems so natural that everyone is self‐deemed knowledgeable and has some expert opinions about it. In fact, cloth has become such an indispensable part of human body and is often referred to as our “second skin.”^1^, ^2^ (Note: In our discussions herein, the terms cloth and apparel are used synonymously, referring to the 3D structure formed by 2D fabrics, whereas textile as a more general term denotes the materials made of fibers (natural or manmade). The term “clothing”, on the other hand, is used to represent the act/status of wearing cloth.)

In order to serve the human body with multifaceted functions, clothes as materials are hierarchal, porous, and flexible with friction‐induced system integrity so as to possess at least the following attributes:^3^, ^4^

-  Flexible and pliable to conform to human body, yet with desirable durability and resistance to various physical impacts for body protection.
-  Soft and smooth to touch but with considerable resistance to abrasion and punctuation.
-  Permeable for body perspiration and skin breathing, while with certain proof to external liquid penetration.
-  Biocompatible with human body, yet chemically active for dying and printing.
-  Light in weight to reduce wearing burden but rigid enough for body shaping and protection.

One of the major functions of clothing is for body protection against various hazards such as environmental, chemical, physical, and hygienic perils, and thermal protection is one of the essentials. Cloth has been with us for thousand years, and we all wear clothes incessantly and keep such intimacy through entire life. Even so cloth has seldom been taken as a materials system for thorough scientific research, as stated by Pan in ref. 3 “It is, therefore, surprisingly puzzling that the materials are so critically indispensable to us and, yet, have been so taken for granted that many rarely pause to think about textiles from a materials science point of view. Textiles, in fact, remain to be poorly understood in terms of materials sciences rigor.”

There have been some books with systematic treatment of the cloth thermal comfort.^2^, ^5^, ^6^, ^7^ However when coming to current research activities in this area, what one may find is the utter rarity of the publications on the topic: typing the key phase “cloth thermal comfort” into the Web of Science generated a mere 23 articles, and very few of them deal with the fundamental principles of clothing, and none of those appears sufficiently comprehensive. Of the 23 papers, one with highest citations^8^ is on an attempt to mimic the wool fiber structure into a manmade fiber, by assembling multiscale fibrils into a tree‐like channel net, thus providing an efficient heat transfer property. Another one^9^ is on the theoretical analysis of water vapor diffusion through porous semipermeable barrier textile material, to evaluate its applicability for sport applications.

One of the major causes of this situation may be attributed to the scientific intricacy of the topic. However incredible it may sound to many including scientists, investigation of the physics pertaining to clothing is rather complex: it involves several subsystems including human body, the surrounding environment, and the multilayered cloth, jointly forming a triad system with such influencing parameters as temperature, humidity, air flow, phase change dynamics, and random causes, on top of the aforementioned complex material behaviors of cloth. This at least partly explains why comprehensive analysis of the clothing process dealing with those complications has rarely been done by researchers.^1^, ^10^

A further alarming sign is from a high profile paper^11^ on clothing thermal comfort published in a prime journal, accompanied by an invited review article^12^ and even an animated video clip. The paper itself dealt with the topic in an overly simplified frame and the analysis conducted is conceptually flawed, as disclosed in an e‐letter to the editor^13^ and detailed here in next section. This exposes an astonishing ignorance about clothing physics, even among some veteran physicists, and thus prompted the initiation of this article.

So this work attempted to uncover several critical yet so far largely ignored issues including, change sign in temperature gradient between body and ambience; the behavior nonaffinity between cloth and its components; cloth as a porous composite, etc. Still it is not intended to be a comprehensive review of the topics, as there are a few books^2^, ^5^, ^6^ and published articles^1^, ^14^ for that. In the discussions below, to maintain focus on the targeted issues, even though there may be various improved forms of the original theories, we only use the one sophisticated enough, not necessarily the most updated/accurate, to facilitate our analysis.

## 2 A Dubious New Cloth

To reveal the hidden issues in clothing thermal comfort, we will first examine and discuss the idea and related flaws in the work by Hsu et al.^11^ of facilitating body heat radiative dissipation using a polyethylene (nanoPE) fabric. For simplicity, they ignored such factors including various heat conductions and convections which will be discussed in a later section here. The nanoporous nanoPE used is a nonwoven piece with specified nanosized holes that, according to the authors,^11^, ^12^, ^15^ allow the infrared radiation from the human body to dissipate through the new fabric easily, but shields the external visible light, thus eliminating the associated incoming heat while making the body visibly opaque to human eyes. The test results indicated that the nanoPE fabric swatch is “2.0 °C lower than a cotton sample.”^11^

Most recently, the same group extended their idea of IR‐transparent nanoPE further and developed a dual‐mode fabric with an asymmetrical (dual) thermal emitter embedded in the asymmetrical thickness of nanoPE so that flipping the fabric will switch between the radiative functions of cooling and heating.^16^ This new extension however inherits the same blunders from the first paper.^11^

Acceding to their assumed validities of existing heat transfer theories to this case, for a piece of fabric swatch in contact with body skin in a steady state and ignoring the convection as in ref. 11 the specific heat flux (energy per time per area) *P* transferred through the swatch is caused by both radiation (*P*_1_) and conduction (*P_2_*) as

$$P=P_{1}+P_{2}$$

In the steady state, the temperature inside the cloth surface equals to the skin temperature *T*_s_, yet the temperature *T* of outside cloth surface can be any point between *T*_a_ ≤ *T* ≤ *T*_s_, depending on the value of the thermal Biot Number of the swatch,^17^ where *T*_a_ is the ambient temperature and *T*_a_ < *T*_s_. In clothing cases at this temperature range, the radiation part is much more dominant,^1^, ^10^ i.e., *P*_1_ >> *P*_2_, we can limit our discussion to thermal radiation as did in ref. 11, adopting the same symbols and formulation originally in ref. 11 so that

(1) $$P\approx P_{1}=\varepsilon \sigma A\left(T_{s}^{4}-T_{a}^{4}\right)$$

where σ in the equation is the Stefan–Boltzmann constant, *A* is the radiating surface area, and ε is the emissivity factor. *T*_s_ is again the skin temperature at =33.5 °C (isothermal) and *T*_a_ is the ambient temperature.

**Figure 1:**
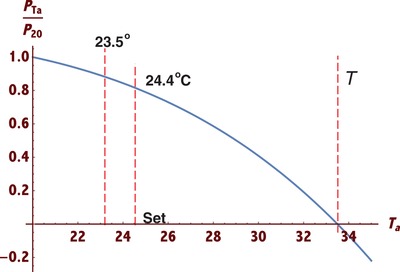
The radiation heat loss as a function of ambient temperature.

A simple plot based on Equation (1) is shown in **Figure** **1**, where for easy comparison the vertical axis represents the relative heat dissipation—the heat dissipation *P* at any given ambient temperature *T*_a_ normalized by the maximum value achieved when the ambient temperature is at the minimum, say *T*_a_ = 20.0 °C. It is clear from the result that at the practically fixed skin temperature *T*_s_, a smaller *T*_a_ (<*T*_s_) will generate a larger *p* value, i.e., a more effective radiation heat release in a cooler environment. This will be more apparent if the thermal conduction *P_2_* is included as it is also a decreasing function of the ambient temperature *T*_a_.

In the study,^11^ the authors took all the measurements at the ambient temperature *T*_a_ = 23.5 °C (74.3 °F). However, at this level, body heat would unlikely be a concern for many people, after all it is still not warm enough for air conditioning (AC) whose set point is nearly 2 °F higher at *T*_a_ = 76.0 °F, according to ANSI/ASHRAE standards.^18^

Once the ambient temperature *T*_a_ is beyond 76.0 °F (24.4 °C), the AC is on and the relative heat dissipation in Figure 1 is reduced, close to 0.70 of that when *T*_a_ = 20.0 °C, versus 0.76 when *T*_a_ = 23.5 °C. So the nanoPE will no longer be 2.0 °C cooler than the cotton at this higher *T*_a_. If the ambient temperature *T*_a_ further increases to around the skin temperature *T*_s_ = 33.5 °C, this nanoPE will completely stop functioning! In other words, once the weather is really hot, exceeding the body temperature, this nanoPE will lose all its cooling power! That is, this nanoPE fabric indeed promotes body chilling, but only in cool days when *T*_a_ < *T*_s_, or it resolves a nonexistent problem.

## 3 The Culprits in the Problem

Upon examining the problems to further expose the blunders in this IR‐transparent nanoPE work, several causes below are found responsible.

### 3.1 Changing Sign of the Temperature Gradient

The problem above shows how little we really understand the physics in clothing, and more importantly, it divulges a rarely noticed yet very critical fact. Note again in Figure 1, once we extend the ambient temperature *T*_a_ along the *x* axis further beyond the skin temperature *T*_s_ = 33.5 °C (92.3 °F) into a hot day, the relative heat dissipation will change its sign and turn into negative, meaning once the ambient temperature exceeds the skin temperature, instead of dissipating body heat to the ambient, this nanoPE approach will induce heat from the ambience to the body. This new clothing would actually exacerbate the scorching of human body in hot days!

Thermal transfers via conduction, convection, and radiation are all temperature gradient dependent. In this case, the temperature gradient is

(2) $$\Delta T=T_{s}-T_{a}$$

As the skin temperature *T*_s_ = 33.5 °C is practically fixed, and the ambient temperature *T*_a_ may be lower (cooler), equal to or greater than (hot) *T*_s_. That is, the sign of the temperature gradient, hence the direction of heat transfer, will be reversed accordingly as the weather gets hot. That is, the entire system will experience a reversal in heat transfer direction around *T*_a_ = *T*_s_.

According to the heat energy conservation equation, a complete form for Equation (1) for a clothed body can be, for steady state and within certain limit, expressed as^19^

(3) P=M−W−E−[Q]

where *P* is the total heat flux; *M* is metabolic rate energy generated by body, always positive; *W* is mechanical work done by body to the ambience, always −*W*; *E* is rate of total latent heat loss due to respiration and sweat evaporation, always –*E*; [*Q*] = total rate of sensible heat loss from skin (dry heat exchange), can be positive or negative as discussed below.

When the total heat flux *P* = 0, the heat body received and dissipated cancelled out so the body is in a steady heat balance; otherwise when *P* > 0, body receiving more heat, or *P* < 0, body losing more heat. Of the parameters on the RHS of Equation (3), the metabolic body rate *M* and the work performed by body *W* remain virtually constant in this study so the body thermal comfort depends only on the remaining two factors; the evaporation of the latent sweat *E*, which is always negative, i.e., sweating always promoting body cooling, and the sensible heat exchange [*Q*]. The signs of [*Q*] follow the sign of the temperature gradient; the positive sign represents the sensible heat from the ambient added to the body, while negative sign is the heat released from the body to the ambient, i.e.

(4) $$[Q]=\begin{array}{c} Q\;\mathrm{if}\;\Delta T>0,\;a\;\mathrm{cool}\;\mathrm{day}\\ -Q\;\mathrm{if}\;\Delta T<0,\;a\;\mathrm{hot}\;\mathrm{day} \end{array}$$

In practice, improving body cooling would be necessary only in hot days when the ambient temperature exceeds the skin temperature, i.e., *T*_a_ ≥ *T*_s_ or Δ*T* < 0, so that [*Q*] = –*Q*, fetching additional heat to the body. Eq. 3 becomes

(3a) P=M−W−E+Q

so that *P* increases. Actions must be taken (e.g., body sweating more to raise −*E* or taking off cloth to reduce +*Q*) to return the body to the original thermal equilibrium. When weather is too hot *T*_s_ << *T*_a_, we have to resort to other external means like a fan or air conditioner. Whereas in cold days

(3b) P=M−W−E−Q

Our body has to lose more body heat via *Q*.

**Figure 2:**
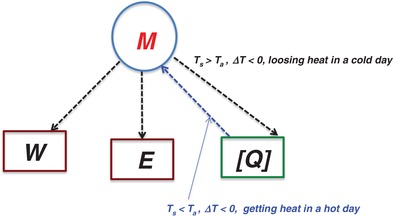
Body heat energy balance and temperature gradient effect.

Stress the subtlety of [*Q*]. In a hot day Δ*T* < 0, so that [*Q*] = –*Q*: the body is gaining more heat in a hot day because of [*Q*]. While in a cold day Δ*T* > 0, so that [*Q*] = *Q*: the body is losing more heat due to [*Q*]. In other words, regardless in winter or summer, except at temperatures close to skin temperature, [*Q*] is always working against body comfort, as illustrated in **Figure** **2**.

The implication of this reversing nature of [*Q*] can be profound; any scheme attempting to alter the cloth thermal comfort by controlling the sensible heat loss [*Q*], i.e., changing the thermal conductivity, convectivity and radiativity, would lead unexpectedly the opposite result. This is exactly the problem when using the nanoPE methodology in ref. 11. Therefore, when assessing the efficacy of fabric thermal properties, the sign of the temperature gradient has to be such that it is consistent with the actual cloth wearing situation.

By the way, among the three sensible heat loss mechanisms, usually heat radiation is by far the most effective.^1^, ^10^ In severe cold weather, e.g., in the arctic area *T*_s_ > *T*_a_, as our body heat is still escaping via the direct heat loss −*Q*, a shield layer made by materials with high irradiativity such as metals is often adopted, preferably as a liner inside the cloth, to retain the body heat. Whereas in a severely hot case *T*_s_ > *T*_a_, e.g., on‐site viewing a live volcano, the temperature gradient dictates the direct heat dumping +*Q* from the ambient to the body. It is desirable to put the metallic sheet outside the cloth to fend off the external heat.

Then it is apparent that the evaporation heat loss *E*, being independent of the temperature gradient, is the only mechanism that provides an indispensable and effective route for body cooling.

### 3.2 The Nonaffinity Phenomenon

Another intriguing thermal phenomenon in clothing, very puzzling for some alert consumers, is the seemingly disconnection between the thermal properties of a cloth and its constituent fibers. The same (e.g., cotton) fibers can be made into a T‐shirt to keep body cool in summer; or into a thick coat to keep body warm in winter, i.e., the thermal properties of the constituent fiber seem to have no connection with the thermal properties of the cloth system. We call this disconnection a nonaffinity^3^ between the behaviors of the system and its constituents.

This problem of the relationships between the microconstituents and the macrosystem is of course typical in statistical physics. The secret here lies in the fact a cloth is a porous composite and the fibers in the cloth are not the only, not even the most essential, element in determining the system thermal performance. Giving the excellent thermal insulation capacity of the air in the pores, the fibers only serve as the medium building those pores. where air stays still and thus dominates the cloth thermal properties. In other words, when coming to cold weather, it is the air in the cloth that keeps our body warm. A more rigorous analysis is given in ref. 20, concluding that our tactilely sensed warmth is actually related to a compound parameter termed thermal effusivity ε, defined as

(5) $$\varepsilon =\sqrt{c_{p}\rho k}$$

where κ is the thermal conductivity, ρ is the specific density, and *c*_p_ is the specific heat capacity of the material. Hence a material with higher effusivity *ε* value will render a warmer touch feeling. For cloths made of the same fibers, the cloth density ρ can vary by several orders of magnitude,^21^, ^22^ while the κ and *c*_p_ values change only mildly. Therefore, we adjust the thermal behaviors of cloth by controlling its density; for the same fiber type, when fabric density is high as in a T‐shirt, it can keep us cool in summer, whereas in winter we wear cloth made of low density fluffy fabric as in a coat to maintain the body warmth. That is, when coming to thermal behavior of the cloth, the air in pores, no longer an unintended entity, plays the dominant role.

Furthermore this nonaffinity in clothing thermal comfort suggests that the primary factor impacting cloth thermal comfort is the structure (or the porosity) of the cloth, and the fiber type plays a negligible role. Such nonaffinity exists only in porous structures not in continuum. For instance, pulling a fiber out of a cloth results in a single fiber that lost all the cloth characteristics, whereas a gold particle from a nugget is still gold.

Further, this nonaffinity exists between any two links along the hierarchy of a cloth system, e.g., therefore the properties of a single fabric swatch tested ex situ may not be taken directly as the properties of a cloth on a human body as did in ref. 11.

Nonetheless, fiber types play important roles in other cases. For example, fiber wicking/wetting properties are type dependent—cloth made of natural fibers are definitely more comfortable. Fiber type will also impact the fabric surfaces which trap different amount of boundary air layer depending upon the roughness of the surface. In addition, it will alter the contact feeling and the surface thermal properties. Furthermore, some of the water from the condensed perspiration will also reside in the fabric, causing the change of fabric thermal conductivity, and leading to reactions such as sorption heat^23^ and the “heat pipe” effect,^24^ depending on fiber types. Some are discussed below.

### 3.3 Fiber Sorption Heat

Another often ignored, nonetheless important mechanism in cloth thermal comfort has to do with the fiber sorption heat. Except in absolutely dry or raining cases, a cloth is a composite formed by fiber, air, and moisture. Consequently, any property of the cloth is a contested result of the contributions from all three constituents. There are two potential sources for moisture, the ambient relative humidity and the body sweating.^25^

When polymeric fibers absorb water, the interactions between polymer chains and water molecules are largely exothermic so that the sorption heat is generated, exerting significant thermal effect on the cloth, as reported by Mordon and Hearle:^23^ “For example, ongoing from an atmosphere of 18 °C, 45% r.h., indoors to one of 5 °C, 95% r.h., outdoors, the regain of wool would change from 10% to 27%. A man's suit, weighing 1.5 kg, would give out 6000 kJ owing to this change, that is, as much heat as the body metabolism produces in 12 h.”

### 3.4 Cloth Fitness, Air Gap Between Cloth Layers and Other Important Factors

Clothing is only physiologically necessary in cold weather when *T*_s_ > *T*_a_, whereas wearing cloth in summer is largely a societal etiquette. Usually we have to wear undergarments in addition, thus introducing the air gap between cloth layers even if the cloth is a perfect fit so that the heat leaking via sides is negligible. As increased air in the interlayer gap will promote thermal insulation,^10^ while the air gap, as well as body movement, will incite convection. These factors influence the system thermal balance drastically. Unfortunately, due to the complex nature of the interlayer air gap between cloth layers and of the various body contour, the air gap scale, distribution, physical state, etc., incorporating this effect into theoretical analysis is still a pending challenge.^26^

### 3.5 Contact Comfort versus Steady Heat Insulation

As mentioned before different fiber types will influence the fabric surface, it will do cause different thermal sensation. This is due mainly to the transient effect, not related to sensible heat loss *Q*. First, in any thermal transfer process, particularly in porous materials like textiles, there are essentially two distinctive stages involved; the transient and the steady stages; the system state including temperature distributions between the two stages are very different.^27^ The study in ref. 11 however failed to differentiate them, e.g., no specification of the stage was given on the data measured. Two different clothes may lead to the same thermal performance at the steady state; still they can cause distinctive transient thermal behaviors and hence the sense of warmth toward the cloth.

## 4 How to do it Right

The supreme question remains on how to appropriately evaluate the efficacy of a new material in improving the cloth thermal comfort. As in dealing with any phenomena, there are different types of method.

### 4.1 Experimental Approach

The first logical experimental approach to assess the efficacy of a new cloth is to actually wear it.^28^, ^29^ Wearing trial by human subject is the most direct. However like in many human sensory processes, the inherent bias in human preference often render the method ineffective.^30^ Also it will not be allowed to employ human subject when the temperature is at extreme levels.

Then full scale manikins have been developed for evaluating the cloth performance including thermal behaviors.^25^, ^29^, ^31^ Yet how to scale the human size and to imitate closely the human metabolic and sweating functions remain to be the challenging issues.

### 4.2 Theoretical Path

Unfortunately, the energy conservation relation in Equation (3) is insufficient, as a sufficient tool for design optimization because of lacking the established relationships between the ambient temperature *T*_a_ and all the thermodynamic variables in the equation. For instance, at given metabolic rate *M*, the performed work *W*, and even the rate of sensible heat loss [*Q*], it is still not clear exactly how the rate of total latent heat loss *E* will change as a function of ambient *T*_a_ and the relative humidity.

**Figure 3:**
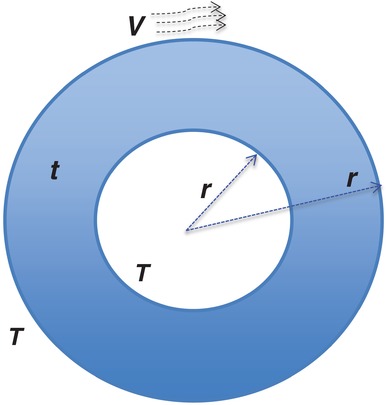
Cross section of heat transfer through a clothed model.

However, we may use some known physical laws to tackle easier or local questions. For instance, consider the sensible heat case only so we know all the physics involved. We can choose the thickness of cloth *t* to minimize the body heat loss in a cold day at a given temperature gradient *T*_s_ > *T*_a_. We assume the body‐cloth system to be represented by a cylinder of the inner (equivalently body) radius *r*_0_ and outer radius *r*_1_ = *r*_0_ + *t* in **Figure** **3**. Then from ref. 17 for steady state and an isotropic system, the total sensible heat lose *Q* per unit length through the cloth of total thickness *t* is the sum of all 3 mechanisms of sensible heat transfer

(6) $$\begin{array}{l} Q\;=\;q_{c}+q_{v}+q_{r}=\Delta T\left(\frac{2\pi k}{\ln\left(\frac{r_{0}+t}{r_{0}}\right)}+2\pi h_{v}{\left(r_{0}+t\right)}^{2}\right)\\ \;\;\;\;\;\;\;+2\pi \varepsilon \sigma {\left(r_{0}+t\right)}^{2}\left(T_{s}^{4}-T_{a}^{4}\right) \end{array}$$

where *k* is the thermal conductivity and *h*_v_ is the cloth surface convectivity at wind speed *V*_a_. The only variable in the equation is the cloth thickness *t* and its critical value *t*_c_ to minimize the heat lose can be derived as in thermal engineering,^17^ i.e., when *t* = *t*_c_, there is

(7) $$\frac{dQ}{dt}=0$$

Furthermore, there are additional considerations related to cloth thermal comfort, such as the cloth mechanical behaviors, fabric finishes, washings, etc. Another additional concern is the interconnections among the thermal properties of cloth: when changing one of them all others may be impacted. For more information, there is a recent review article on the complex and restrictive requirements for cloth comfort.^4^

The fundamental objective of research on the triad system of body‐cloth‐ambience, as stated by Parsons,^19^ is to establish “Thermal models integrate the principles of heat transfer, heat balance, thermal physiology and thermoregulation along with anthropometry and anatomy into a mathematical representation of the human body and its thermoregulatory systems. When presented on a computer, these ‘complete' models can simulate the thermal responses of a person and provide a prediction of the dynamic response of the body to any environment.”

So far we have accumulated some knowledge about the human thermoregulation,^6^, ^19^, ^32^ for instance, the sweating rate of human body can reach as high as 2 kg h^−1^ for a short period and 0.9 kg h^−1^ over several hours. Also it can be easily calculated that a human body of normal size would have roughly 2 m^2^ total surface area, with normal 90 W m^−2^ heat production capacity, yet only 0.25 kg h^−1^ sweat is needed to dissipate all the body heat generated.^6^, ^19^, ^32^ Therefore, the evaporation of the sweat has the potential to sufficiently dissipate the heat released from the human body to maintain the cloth heat balance^32^ of course at normal ambient conditions.

On the other hand, what we can present so far are almost all the terminal or extreme values. To describe in detail the dynamics and stochastics in this complex triad system is still far beyond our reach, even though we have identified all the effective parameters in the heat exchange process, and have available of all the general physical and mathematic laws and principles. Still we know, to just name a few, very little the dynamics of sweat rate *E* change as a function of the ambient temperature *T*_a_ and sensible heat loss rate *Q*, including both temporal and spatial distributions; very little how the changes in sweat rate *E* will in turn alter the properties of the cloth (moisture condensation and permeation); let alone the interperson and intraperson body variations in thermal physiology and thermoregulation along with anthropometry and anatomy.

Challenges are daunting but there are reasons to be optimistic, chiefly that because of the arising interests to wearable technology, unprecedented efforts from industry and academia, especially nontextile fields, starting to focus on clothing science. Needless to say, as dealing with any complex problem, simplification is a necessary initial step: but it has to be done correctly. To start, a sufficiently clear understanding of the problem has to be achieved so as to decide what/how the simplification should be done.

## 5 Conclusions

In order to serve human body with multifaceted functions, clothes as materials are hierarchal, porous, and flexible with friction‐induced system integrity. To understand clothing thermal comfort, one has to tackle the complex interactions between human body, cloth, and the surrounding ambience.

Clear distinction and connection between the microconstituents and the macro system have to be established. There is a nonaffinity between the behaviors of the system and its constituents and the properties of a single fabric swatch tested ex situ may not be taken as the corresponding properties of a cloth. This nonaffinity in clothing thermal comfort suggests that the primary factor impacting cloth thermal comfort is the structure (or the porosity) of the cloth, rather than the fiber type.

Attention has to be paid to the changing sign of the temperature gradient Δ*T* between body and the ambience. In a hot day Δ*T* < 0, the body is gaining more heat. While in a cold day Δ*T* > 0, the body is losing more heat. In other words, regardless the weather, sensible heat transfers are always working against body comfort. Therefore, when assessing the efficacy of fabric thermal properties, the sign of the temperature gradient exerted has to be consistent with the cloth wearing situation.

Fiber types are important in cloth wicking/wetting properties, the fabric surfaces, the transient cloth contact feeling and fiber sorption related phenomena. There are other important factors including the natural and forced convections, due to air gap between cloth layers, and body movement.

Finally, when handling a complex problem like this, certain simplifications are necessary. Nonetheless an appropriate picture of the entire system physics has to be developed first so that such simplification can be done correctly.

## Conflict of Interest

The authors declare no conflict of interest.
